# Discovery of a ROCK inhibitor, FPND, which prevents cerebral hemorrhage through maintaining vascular integrity by interference with VE-cadherin

**DOI:** 10.1038/cddiscovery.2017.51

**Published:** 2017-08-21

**Authors:** Shang Li, Nana Ai, Mingyun Shen, Yuanye Dang, Cheong-Meng Chong, Peichen Pan, Yiu Wa Kwan, Shun Wan Chan, George Pak Heng Leung, Maggie Pui Man Hoi, Tingjun Hou, Simon Ming-Yuen Lee

**Affiliations:** 1 State Key Laboratory of Quality Research in Chinese Medicine, and Institute of Chinese Medical Sciences, University of Macau, Macau, China; 2 College of Pharmaceutical Sciences, Zhejiang University, Hangzhou, China; 3 Institute of Functional Nano and Soft Materials (FUNSOM), Soochow University, Suzhou, China; 4 School of Biomedical Sciences, Faculty of Medicine, The Chinese University of Hong Kong, Hong Kong, China; 5 State Key Laboratory of Chinese Medicine and Molecular Pharmacology, Department of Applied Biology and Chemical Technology, The Hong Kong Polytechnic University, Hong Kong, China; 6 Pharmacology and Pharmacy, Faculty of Medicine, The University of Hong Kong, Hong Kong, China

## Abstract

Hemorrhagic stroke occurs when a weakened vessel ruptures and bleeds into the surrounding brain, leading to high rates of death and disability worldwide. A series of complex pathophysiological cascades contribute to the risk of hemorrhagic stroke, and no therapies have proven effective to prevent hemorrhagic stroke. Stabilization of vascular integrity has been considered as a potential therapeutic target for hemorrhagic stroke. ROCKs, which belong to the serine/threonine protein kinase family and participate in the organization of actin cytoskeleton, have become attractive targets for the treatment of strokes. In this study, *in vitro* enzyme-based assays revealed that a new compound (FPND) with a novel scaffold identified by docking-based virtual screening could inhibit ROCK1 specifically at low micromolar concentration. Molecular modeling showed that FPND preferentially interacted with ROCK1, and the difference between the binding affinity of FPND toward ROCK1 and ROCK2 primarily resulted from non-polar contributions. Furthermore, FPND significantly prevented statin-induced cerebral hemorrhage in a zebrafish model. In addition, *in vitro* studies using the xCELLigence RTCA system, immunofluorescence and western blotting revealed that FPND prevented statin-induced cerebral hemorrhage by enhancing endothelial cell–cell junctions through inhibiting the ROCK-mediated VE-cadherin signaling pathway. As indicated by the extremely low toxicity of FPND against mice, it is safe and can potentially prevent vascular integrity loss-related diseases, such as hemorrhagic stroke.

## Introduction

Hemorrhagic stroke, which accounts for 20% of all strokes, occurs when a weakened vessel ruptures and bleeds into surrounding brain tissues. The accumulated blood (also referred to as hematoma) compresses and damages the surrounding brain.^1^ Hemorrhagic strokes have been treated by anticoagulants, antihypertensives and antiplatelets through controlling high blood pressure and/or managing atrial fibrillation in high-risk patients.^2,3^ Loss of the vascular endothelial integrity leads to the rupture of vessels and blood flow into interstitial spaces. For instance, as a common vascular dysplasia of cerebral hemorrhage, cerebral cavernous malformation (CCM) is caused by loss of the vascular endothelial integrity.^4^ CCM can potentially be treated with ROCK inhibitor to reverse vascular leak.^5^ Therefore, intracerebral hemorrhage (ICH) may be prevented by maintaining the vascular endothelial integrity.

As a powerful model system, zebrafish has been widely used to unravel the basic genetic and cellular mechanisms of cerebrovascular diseases.^6^ ICH in zebrafish embryos can be easily and directly observed, thus allowing rapid screening of a huge number of mutagenized, preventive or therapeutic compounds for hemorrhage defects. Statins are a class of drugs used to lower high cholesterol levels and to prevent associated complications, for example, by treating cardiovascular diseases through inhibiting HMG-CoA reductase. However, statins have been associated with an increased risk of ICH.^1,7,8,9,10^ Atorvastatin can induce cerebral hemorrhage in zebrafish through loss of vascular stability in the brain.^11^ In addition, it induces the rupture of cerebral vessels by undermining the establishment of endothelial cell-to-cell associations.^12^ As discussed above, and given the molecular mechanisms of vascular development in vertebrates, zebrafish is a useful *in vivo* model for studying vascular integrity.

As serine/threonine kinases, ROCK1/2 contribute to the formation of stress fibers by inactivating myosin phosphatase and phosphorylating myosin light chain (MLC), which regulates the assembly of stress fibers. By regulating the contractility of endothelial cells (ECs), pMLC has a crucial role in vascular tone and functions. In addition, ROCK activates Lim kinase, suppresses cofilin, prevents actin depolymerization and elevates contractility by phosphorylating MLC directly.^13^ Increased contractility disrupts cell–cell adhesion and improves vascular permeability. Therefore, ROCK inhibitors can relieve CCM and vascular leakage by enhancing endothelial cell–cell junctions.^5^

**Figure 1:**
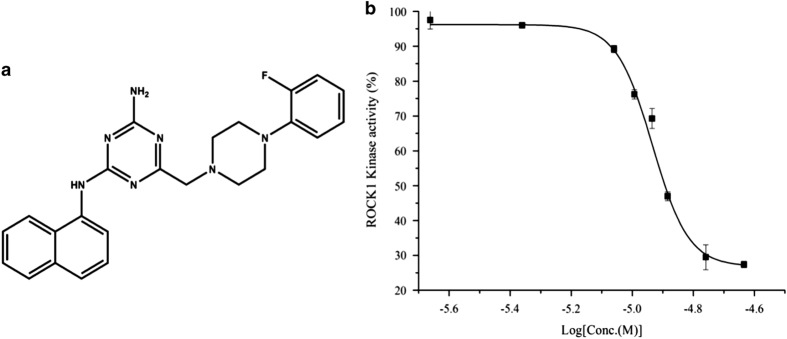
(**a**) The structure of FPND. (**b**) Concentration-dependent inhibition of ROCK1 kinase activity by FPND.

Virtual screening based on molecular docking has become a powerful strategy for identifying lead compounds.^14^ The high-resolution X-ray structure of ROCK1 provides a basis for structure-based drug design. Our group has initiated a research program to identify new drug candidates targeting ROCK for the prevention of hemorrhagic stroke, which combined docking-based virtual screening with a zebrafish model.^15,16^ In this study, we identified a new ROCK1 inhibitor 6-[4-(2-fluorophenyl)-1-piperazinyl]methyl-*N*-1-naphthyl-1,3,5-triazine-2,4-diamine (FPND, PubChem CID: 17168340, chemical structure in Figure 1), which exerted promising protective effects on atorvastatin-induced cerebral hemorrhage in zebrafish *in vivo* and the rupture of endothelial cell–cell junctions in human umbilical vein cells (HUVECs) *in vitro*.

## Results

### Inhibition and binding characteristics of FPND

There are two isoforms of ROCK (ROCK1 and ROCK2), which share the same downstream effector proteins.^17^ The kinase inhibitory assay showed that the IC_50_ value of FPND against ROCK1 was 11.2±2.2 *μ*M (Figure 1), whereas no inhibitory activity was observed for ROCK2. Thus, FPND inhibited ROCK1 specifically.

In order to understand the underlying mechanism, molecular docking, molecular dynamic (MD) simulations and free energy calculations were used to analyze interactions between FPND and the ATP-binding sites of ROCK1 and ROCK2. FPND was docked into the active sites of ROCK1 and ROCK2, and the conformations with the best docking scores were submitted for 5 ns MD simulations and MM/GBSA rescoring. As displayed in Supplementary Figure S1, the ROCK1/FPND complex becomes stable after ~500 ps, whereas the ROCK2/FPND complex reaches stability after 3 ns. Therefore, 100 snapshots, evenly extracted from 3 to 5 ns, were used for the following MM/GBSA binding free energy calculation and decomposition.

The predicted binding free energies of the ROCK1/FPND and ROCK2/FPND complexes are listed in Table 1. The substantial difference in Δ*G*_pred_ (15.69 kcal/mol) indicates that FPND interacts with ROCK1 much more strongly than with ROCK2, which is in good agreement with the experimental data. The non-polar interaction (Δ*E*_vdw_+Δ*G*_SA_=−56.33 kcal/mol) for the ROCK1/FPND complex evidently exceeds that (Δ*E*_vdw_+Δ*G*_SA_=−40.04 kcal/mol) for the ROCK2/FPND complex. However, the polar interactions for these two complexes (Δ*E*_ele_+Δ*G*_GB_=5.77 and 5.15 kcal/mol for ROCK1 and ROCK2, respectively) are similar. Therefore, non-polar interactions have a significant role in determining the specificity of FPND to ROCK1 and ROCK2.

**Figure 2:**
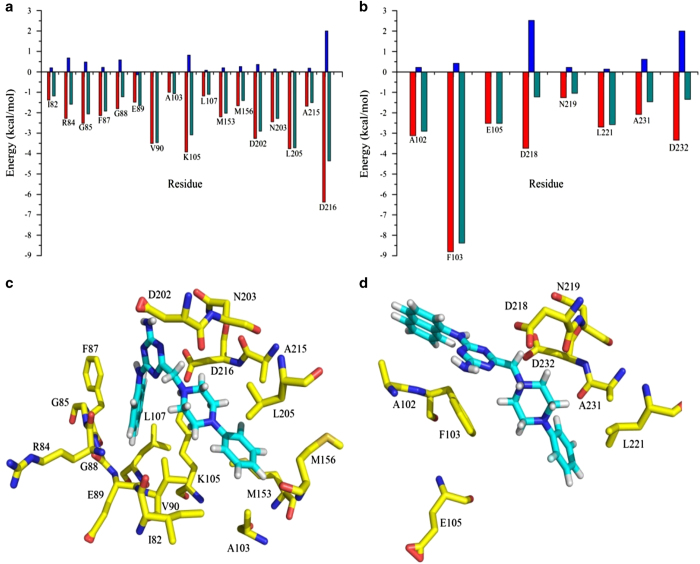
Contributions of the important residues for binding of FPND with (**a**) ROCK1 and (**b**) ROCK2; the red columns represent the non-polar contributions, the blue columns represent the polar contributions and the green columns represent the total energy of each residue. The structures of (**c**) the ROCK1/FPND complex and (**d**) the ROCK2/FPND complex; the carbon atoms of FPND are colored in cyan and the carbon atoms of the key residues are colored in yellow.

In order to determine the most important residues for the binding specificity of FPND, residue–inhibitor interaction profiles were obtained by performing binding free energy decomposition analysis. According to Figure 2, the number of important residues for the binding of FPND to ROCK1 exceeds that for ROCK2. The interactions of FPND with residues Ile82, Arg84, Gly85, Gly88, Val90, Ala103, Lys105, Leu107, Met153 and Met156 in ROCK1 make crucial contributions to the selectivity of FPND. These residues can be roughly divided into two groups: one group includes residues around the naphthalene ring and the other includes residues surrounding the fluorobenzene ring. Residues Ile82, Arg84, Gly85, Gly88, Val90, Ala103, Lys105 and Leu107, especially Val90 and Lys105, form strong non-polar interactions with the naphthalene ring. The contributions of Val90 and Lys105 (−3.46 and −3.08 kcal/mol) are particularly significant, and are dominated by the van der Waals and non-polar desolvation terms (−3.50 and −3.92 kcal/mol). Furthermore, there are strong non-polar interactions between the fluorobenzene ring and residues Met153 and Met156. The contributions of Met153 and Met156 are −2.02 kcal/mol and −1.4 kcal/mol, respectively. In summary, interactions with residues Val90, Lys105, Met153 and Met156 enhance the binding affinity of the ROCK1/FPND complex.

### FPND prevented atorvastatin-induced cerebral hemorrhage in zebrafish embryos

**Figure 3:**
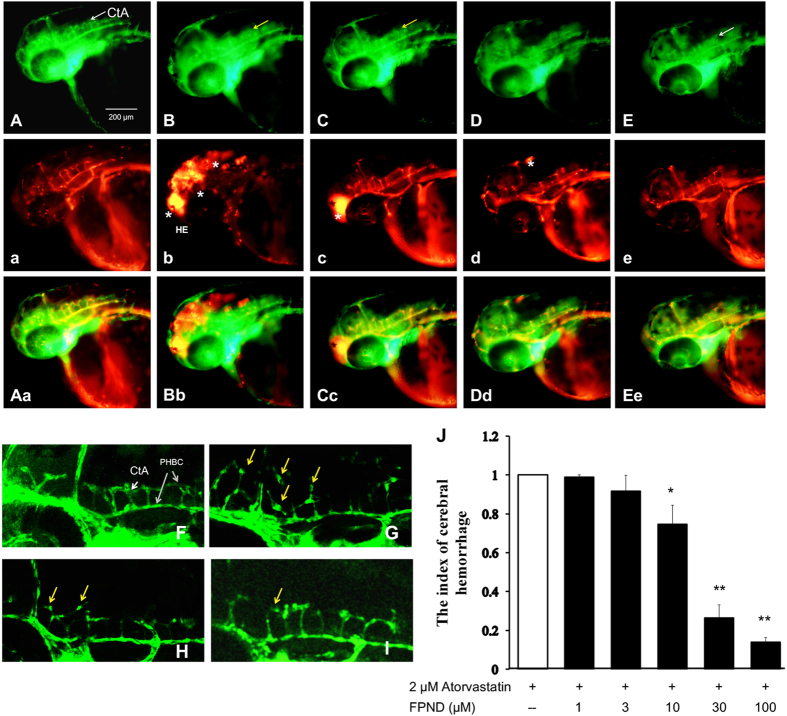
The preventive effect of FPND against atorvastatin-induced cerebral hemorrhage in developing zebrafish. The 22 dpf embryos were pretreated with either 0.1% DMSO (**A**, **B**, **F** and **G**), 10 (**C **and **H**), 30 (**D** and **I**) or 100 *μ*M (**E**) FPND for 3 h and replaced with 0.1% DMSO (**A** and **F**) or 2 *μ*M atorvastatin (**C**–**E** and **G**–**I**) for 24 h. (**A** and **F**) The embryos treated with 0.2% DMSO (solvent) served as the normal control group. At 48 hpf, a lateral view of the hindbrain of the wild-type embryo shows CtA (white arrows) draining into the PHBC. Homozygous double transgenic zebrafish *Tg (fli1a: EGFP) y1 and Tg (gata1: dsRed) sd2*; the green fluorescence is *Tg (fli1a: EGFP) y1* (**a**–**e**), the red fluorescence is *Tg (gata1: dsRed) sd2* (**A**–**E**), and the third column is the overlapping photo of the first two columns (**A**a, **B**b, **C**c, **D**d and **E**e). The asterisks indicate erythrocyte accumulation in the cerebral hemorrhage region of the zebrafish head. The yellow arrows indicate the morphologically abnormal blood vessels. White scale bar=200 *μ*m. (**J**) The representative index of hemorrhage indicates that FPND could prevent atorvastatin-induced cerebral hemorrhage in zebrafish in a dose-dependent manner. Data presented in the bar graphs are the mean±S.D. of three independent experiments. *P<0.05 and **P<0.01 (*versus* control group) were considered significantly different.

We have previously reported that ROCK inhibitors protected against statin-induced cerebral hemorrhage.^15,16^ We established a *Tg (fli1: EGFP) y1* and *Tg (Gata1: dsRed)* double transgenic zebrafish model to test the protective effects against statin-induced cerebral hemorrhage of a new ROCK1 inhibitor, FPND. In this study, 1 dpf embryos were treated with 2 *μ*M atorvastatin for 24 h. Hemorrhages were identified through o-dianizidine staining of erythrocytes (Supplementary Figure 2) and fluorescence imaging of blood vessels and erythrocytes in *Tg (fli1: EGFP) y1* and *Tg (Gata1: dsRed)* double transgenic embryos (Figure 3), aiming to observe blood accumulated through leakage in the cranial region (Figure 3B, Supplementary Figure 2B). This hemorrhage symptom was mitigated by pretreatment with FPND dose-dependently (10, 30 and 100 *μ*M) for 3 h (Figures 3C–E, Supplementary Figures 2C–E). To determine whether the observed protective effect was atorvastatin specific, we also exposed zebrafish to pravastatin, another statin drug. Treatment with 10 *μ*M pravastatin for 24 h significantly induced cerebral hemorrhage in 2 dpf zebrafish embryos. FPND also prevented cerebral bleeding in this case (data not shown). In short, FPND prevented statin-induced cerebral hemorrhage, which was activated via the statin-dependent signaling pathway.

### Structure–activity relationship of FPND analogs against atorvastatin-induced cerebral hemorrhage

**Figure 4:**
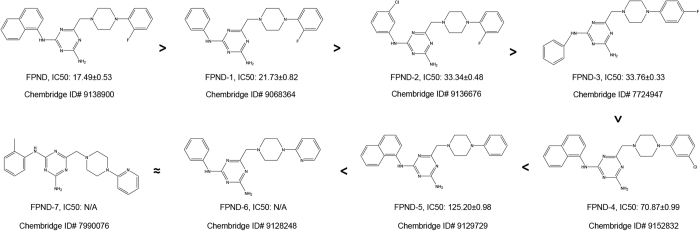
IC_50_ of the FPND analogs to atorvastatin-induced cerebral hemorrhage in zebrafish embryos.

The structure of FPND is mainly composed of naphthalene, triazine and phenylpiperazine. In order to identify the dominant scaffold of FPND contributing to the protective effects, we carried out a substructural search for FPND and obtained seven analogs from the ChemBridge chemical library (Figure 4). According to the experimental data, FPND exhibited the highest activity against cerebral hemorrhage in zebrafish (IC_50_=17.49 *μ*M). The substitution pattern decreased the activity, and the naphthalene ring was crucial for maintaining the activity of FPND. Notably, phenylpiperazine was also required for the anti-hemorrhagic activity (FPND, FPND-1, FPND-2, FPND-3 FPND-4 and FPND-5, *versus* FPND-6 and FPND-7). With a site-changed F atom, FPND-3 was less active than the structurally related FPND-1. Besides, the structures of FPND-4 and FPND-5 resembled that of FPND, but their inhibitory effects were significantly different. Loss of the *F* atom in the phenyl group decreased the activity significantly (FPND *versus* FPND-4 and FPND-5). SAR analysis revealed that the fragments of naphthalene and phenylpiperazine, particularly the F atom of phenylpiperazine, predominantly controlled FPND activity in an independent manner.

### FPND prevented atorvastatin-induced abnormal vascular phenotype

Atorvastatin can cause vascular rupture, followed by bleeding, in the central arteries (CtA) of developing zebrafish embryos.^18^ To gain more insights into the preventive effects of FPND on atorvastatin-induced bleeding, we observed changes in the blood vessels in CtA of atorvastatin-treated *Tg(fli1:EGFP)y1* zebrafish by laser scanning confocal microscopy. The ECs and blood vessels in CtA and the primordial hindbrain channel (PHBC) were well connected and had intact shapes. However, in the atorvastatin-treated group, ECs shrank, accompanied by larger individual EC areas and disconnection between blood vessels in CtA and PHBC (Figures 3G–I). Accordingly, pretreatment with FPND significantly reversed atorvastatin-induced vascular defects. These results indicated that FPND exerted clear protective effects against cerebral hemorrhage, possibly by enhancing the poor contact between ECs.

### FPND prevented the cell–cell junction disruption caused by atorvastatin

**Figure 5:**
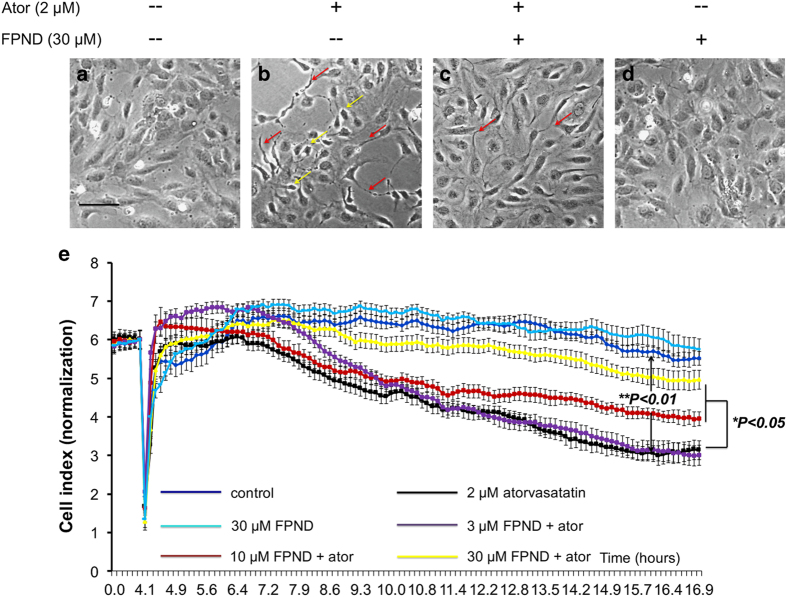
FPND significantly prevented atorvastatin-induced cell retraction and rupture of cell–cell junctions on HUVECs. HUVECs were treated with 0.1% DMSO (**a **and **b**) or 30 *μ*M FPND (**a** and **b**) for 2 h, followed by washout and incubation with 0.1% DMSO (**a** and **d**) or 2 *μ*M atorvastatin (**b **and **c**) for 12 h. Imaging was done with a phase contrast microscope. Yellow arrows show retracted cells; red arrows show formation of pseudopodia. Scale bar=100 *μ*m (black color). (**e**) The representative cell index showed that FPND inhibited atorvastatin-induced EC contraction and rupture of cell–cell junctions. HUVECs were cultured on the E-Plate in complete medium for 48 h and pretreated with 3, 10, 30 *μ*M FPND for 2 h, followed by washout and incubation with 2 *μ*M atorvastatin for 24 h. ‘Ator’ in figure indicates 2 *μ*M atorvastatin. Data presented in the bar graphs are the mean±S.D. of three independent experiments. *P<0.05 and **P<0.01 (*versus* the atorvastatin-alone group) were considered significantly different.

As atorvastatin can cause blood vessel bursting during the formation of CtA, ECs contact poorly.^18^ Further studies showed that 24 h of treatment with 2 *μ*M atorvastatin induced marked changes in the morphology of ECs.^19,20^ We hypothesized that atorvastatin caused poor contact between ECs by inducing cell morphology changes, leading to abnormal morphology of blood vessels, bursting and cerebral hemorrhage. We therefore applied HUVECs as an *in vitro* model to investigate the effects of atorvastatin on endothelial cell–cell junctions. Under normal conditions, HUVECs formed a monolayer of cobblestone-like oval cells that were in close contact along the entire cell periphery (Figure 5a). In contrast, treatment by atorvastatin resulted in shrinkage, with increased formation of pseudopodia of HUVECs from the neighboring ones (Figure 5b). We pretreated HUVECs with FPND for 2 h, and thereafter treated them with 2 *μ*M atorvastatin for 12 h. FPND was able to prevent the changes in cell morphology induced by atorvastatin treatment (Figure 5c). The xCELLigence RTCA system is a non-invasive and label-free platform. HUVECs can be cultured and maintained on an E-Plate to form a stable monolayer, and changes in cell–cell contact can be quantified by measuring the impedance changes across the cell monolayer. As a result, cell–cell interaction, transient contractions and cell layer permeability can be indirectly measured.^21,22,23^ The cell–cell junctions of HUVECs were significantly disrupted by atorvastatin, as shown by the decreased cell index after application of 2 *μ*M atorvastatin. However, pretreatment with FPND prevented the loss of atorvastatin-induced cell–cell junctions in a concentration-dependent manner (Figure 5e).

### FPND prevented atorvastatin-induced loss of cell–cell junctions by regulating actin cytoskeleton and junction protein distribution

**Figure 6:**
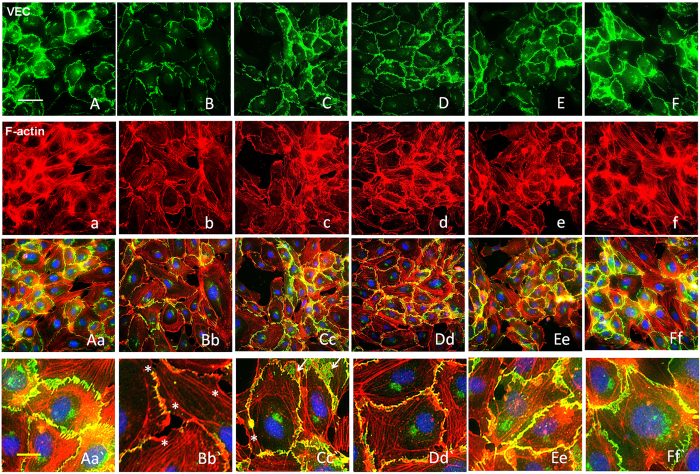
FPND prevented atorvastatin**-**induced VEC junction dissociation and loss of VEC from cell borders. HUVECs monolayers were treated with 0.1% DMSO (**A**, a, Aa, A`; **B**, b, Bb and B`), and 5 (**C**, c, Cc and Cc`), 10 (**D**, d, Dd, Dd`), and 20 (**E**, e, Ee, Ee`; **F**, f, Ff and Ff`) *μ*M FPND for 2 h, followed by washout and treatment with 0.1% DMSO (**A**, a, Aa, A`; **F**, f, Ff and Ff`) or 2 *μ*M atorvastatin (**B**, b, Bb and B`; **C**, c, Cc and Cc`; **D**, d, Dd and Dd`; **E**, e, Ee and Ee`) for 12 h; 0.1% DMSO treatment for 12 h was the vehicle control (**A**, a, Aa, Aa`). Treatment with FPND alone (**F**, f, Ff and Ff`) resulted in a slight decrease in stress fiber formation but with no effect on VEC distribution or cell–cell junctions. The VEC signal was labeled with VEC-specific antibody in green color (**A**–**F**). F-actin was labeled with tetramethyl rhodamine isothiocyanate (TRITC)-phalloidin in red color and nuclei were labeled with the nuclear-specific dye Hochest 33342 in blue color (**A**–**F**). White asterisks indicate formation of scrambled knots, membrane ruffle and focal adhesion complexes. White arrows show a drastic loss of VEC from cell borders and formation of a net-like structure. White and yellow scale bars=50 and 10 *μ*m, respectively.

Adherens junctions (AJs) are involved in the establishment and maintenance of cell–cell adhesion, intracellular signaling and remodeling of the actin cytoskeleton. Cell–cell adhesion, which is mediated by junctional protein VE-cadherin, is the transmembrane component of endothelial AJ and constitutes an intercellular junctional complex, having a crucial role in defining the physiological functions of cells.^24^ VEC functions through the interaction of its cytoplasmic tail with cytoplasmic proteins called catenins. The resulting catenin complexes (VE-catenin, *β*-catenin and p120-catenin) in the cell periphery have key roles in establishing AJs in ECs. To further evaluate the effects of atorvastatin on endothelial cell–cell junctions, immunofluorescence staining with antibodies directed against VE-catenin, *β*-catenin and catenin *δ*-1 (p120-catenin) was conducted, showing abundant distribution of catenins (VE-catenin, *β*-catenin and p120-catenin) at the cell periphery of normal HUVECs (Figures 6A-Aa, Supplementary Figures 3A-Aa and 4A-Aa). Treatment with atorvastatin resulted in VEC junction dissociation, formation of net-like VEC and drastic losses of VE-catenin, p120-catenin and *β*-catenin from cell borders (Figure 6B, Supplementary Figures 3B and 4B). However, the losses were significantly reversed after pretreatment with FPND (Figure 6E, Supplementary Figures 3E and 4E).

Catenins, such as *β*-catenin and p120-catenin, worked in concert to associate the cadherin complex with the actin cytoskeleton. These catenins have essential roles in attaching cadherin to the actin cytoskeleton, which is required for the formation of a restrictive monolayer and the maintenance of cell–cell junctions.^25^ The stability of AJs is mainly dependent on the dynamics of peripheral actin cytoskeleton. HUVECs were stained with rhodamine–phalloidin to visualize F-actin. Normal HUVECs had parallel bundles of F-actin stress fibers. Treatment with atorvastatin induced loss of actin stress fibers, with increased scrambled actin knots and focal adhesion complexes, as well as membrane ruffle formation close to the cell–cell contacts (Figure 6B, Supplementary Figures 3b and 4b). Thus, we hypothesized that atorvastatin disrupted the actin cytoskeleton in response to loss of VEC-catenin, *β*-catenin or p120-catenin from cell borders. Interestingly, the negative outcomes caused by atorvastatin were reversed by FPND pretreatment (Figures 6C–E, Supplementary Figures 3c–e and 4c–e). Thus, the cell border distribution of VEC/catenin complex-dependent reorganization of actin cytoskeleton may participate in the prevention of atorvastatin-induced rupture of cell–cell junctions.

### FPND prevented the rupture of cell–cell junctions by inhibiting the activation of ROCK/VEC signaling pathways

**Figure 7:**
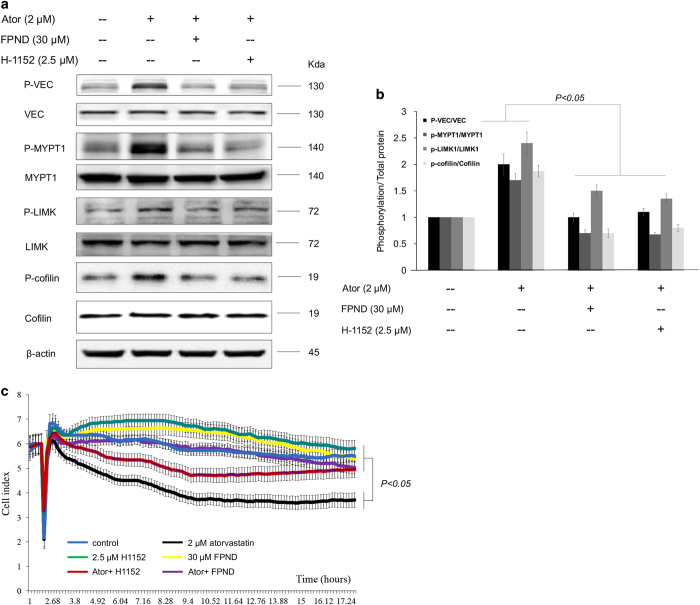
FPND or ROCK inhibitor inhibited atorvastatin-induced ROCK signaling pathways. HUVEC cells were pretreated with either 20 *μ*M FPND or 2.5 *μ*M H1152 for 1 h and then stimulated with 2 *μ*M atorvastatin for 30 min. (**a**) The expression ratios of phosphorylated MYPT1/total MYPT1, phosphorylated LIMK1/total LIMK1, and phosphorylated confilin/total confilin were detected by western blotting with specific antibodies, as indicated; (**b**) the data were quantified by the ratio of the band intensity. (**c**) Inhibition of ROCK significantly prevented atorvastatin-induced rupture of cell–cell junctions on ECs. HUVECs were cultured on the E-Plate in complete medium for 48 h and pretreated with 30 *μ*M FPND or 2.5 *μ*M H1152 for 2 h, followed by washout and incubation with 2 *μ*M atorvastatin for 24 h. ‘Ator’ in figure indicates 2 *μ*M atorvastatin. Data presented in the bar graphs are the mean±S.D. of three independent experiments. *P<0.05, **P<0.01 and ***P<0.005 (*versus* the atorvastatin-alone group) were considered significantly different.

The signaling pathways of ROCK/MYPT1/MLC2 and ROCK/LIMK/cofilin are essential in the assembly of cytoskeleton stress fibers and cell adhesion.^26^ H1152 is a selective ATP-competitive inhibitor of ROCK-I/II.^27^ In order to further confirm that FPND acted as a ROCK inhibitor in ECs, HUVECs were pretreated with either 30 *μ*M FPND or 2.5 *μ*M H1152 for 1 h and then stimulated with 2 *μ*M atorvastatin for 30 min. It is well known that MYPT1 and LIMK1 are downstream effector proteins of ROCK, which can be phosphorylated and activated by ROCK.^28^ We found that once HUVECs were pretreated with FPND, the phosphorylation of MYPT1 and LIMK1 stimulated by atorvastatin was inhibited significantly (Figures 7a and b). In addition, FPND or H1152 treatment also inhibited the phosphorylation of cofilin, an actin-binding protein regulated by LIMK1 (Figures 7a and b). These results showed that FPND was a ROCK inhibitor similar to H1152 and suppressed atorvastatin-induced ROCK activation.

To explore whether the ROCK signaling pathway was correlated with cell–cell junctions in statin treatment, HUVECs were pretreated with FPND, H1152 or ROCK-I siRNA. As shown in Figure 7c, pretreatment with FPND or H1152 prevented the loss of atorvastatin-induced cell–cell junctions. Furthermore, atorvastatin induces VEC phosphorylation in HUVEC cells, whereas FPND, H1152 and ROCK-I siRNA block this effect (Figures 7a and b, Supplementary Figure 5). Moreover, when embryos were pretreated with H1152 for 3 h (Supplementary Figure 6), they died upon 10 *μ*M treatment (data not shown). Below this concentration, atorvastatin-induced cerebral hemorrhage was ameliorated, but the prevention effects were not as strong as those of FPND. These results strongly implied that FPND prevented atorvastatin-induced cerebral hemorrhage via ROCK/VEC signaling pathways.

### FPND was a promising candidate for the prevention of hemorrhagic stroke

**Figure 8:**
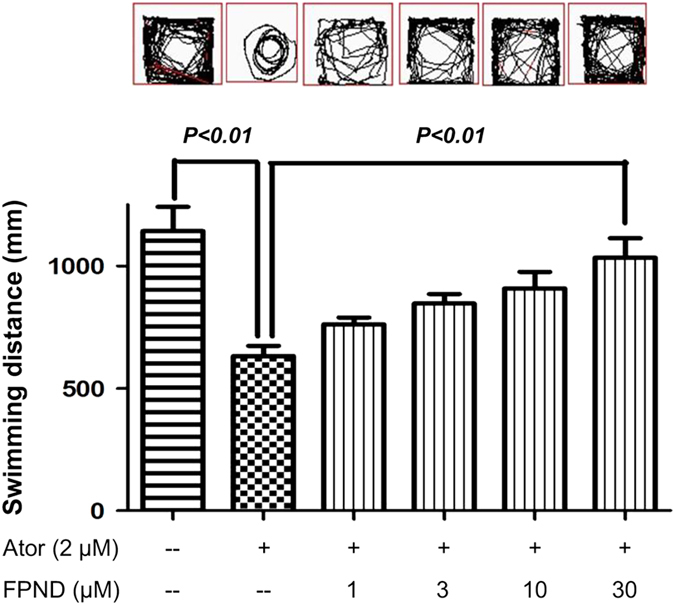
FPND prevented any atorvastatin-related decrease in zebrafish locomotion activity in a dose-dependent manner. In all, 1 dpf zebrafish were treated with or without FPND for 3 h, and then incubated with 2 *μ*M atorvastatin for 3 days. The swimming distances of fishes were examined by the Viewpoint Zebrabox system (Viewpoint, Paris, France) and the total distance moved in 10 min was calculated. Representative patterns of zebrafish locomotion traced from different treatment groups. Statistical analysis of the total distance traveled by each zebrafish larva in the different treatment groups (12 fish larval per group from three independent experiments). The results show the mean distance traveled by 36 larvae and are expressed in mm/10 min. Data presented in the bar graphs are the mean±S.D. of three independent experiments.

The pathophysiology of ICH is complex. Hemorrhage into the brain initially and obviously compresses the adjacent microvasculature by producing hematoma. Intracranial hematoma induces degeneration and necrosis of neurons and the brain. Indexing neurological deficits is important for not only patients with stroke, but also in animal models of ICH.^29^ For zebrafish, such deficits can be characterized using different paradigms associated with complex behaviors such as memory and anxiety, as well as neuroprotection of dopaminergic neurons and movement disorders. In our experiment, 6 dpf zebrafish treated with atorvastatin continuously for 5 days from 1 dpf were subjected to behavioral testing. FPND prevented atorvastatin-induced cerebral hemorrhage in zebrafish embryos. Meanwhile, 6 dpf zebrafish larvae were also subjected to behavioral testing. Atorvastatin significantly decreased their swimming distance, but pretreatment with FPND reversed atorvastatin-induced locomotor activity defects in a dose-dependent manner (Figure 8).

Finally, we tested the toxicity of FPND by orally administering FPND to 10 mice at 10 mg/kg body weight. Meanwhile, another 10 mice were intravenously injected with 0.5 mg/kg FPND. All the mice were alive and the body weights remained stable over 14 days. The calculated LD_50_ of orally administered FPND was higher than 5 mg/kg, and that of intravenously administered FPND was higher than 0.5 mg/kg, suggesting that FPND was safe and worthy of further study.

## Discussion

We herein presented a new ROCK inhibitor for the prevention of cerebral hemorrhage by maintaining vascular integrity. Similar to our results, ROCK inhibitor has been reported to protect against the chronic hemorrhage of CCM lesions in mouse models.^30,31^ However, the detailed mechanism is still unknown. We postulated that ROCK inhibitor had a direct role in maintaining the integrity of blood vessels through inhibition of VEC activities.

Inhibition of the ROCK signaling pathway can relieve both CCM and vascular leak by enhancing endothelial cell–cell junctions.^5^ Activation of ROCK induces actin cytoskeletal rearrangement and disrupts VEC-mediated intercellular adhesion.^32^ Our group has initiated a research program to identify new drug candidates targeting ROCK for the prevention of hemorrhagic stroke, which combined docking-based virtual screening with a zebrafish model.^15,16^ We demonstrated that ROCK1 inhibitors effectively inhibited the phosphorylation of downstream targets in the ROCK signaling pathway *in vitro*, and protected against statin-induced cerebral hemorrhage *in vivo*.^15,16^ The barrier function is maintained and regulated by AJs connecting ECs and neighboring cells. As the most important cell adhesion protein in endothelial AJs, VEC ensures that ECs stay connected and limits leakage from blood vessels.^33^ VEC interacts with catenins (for example, p120-catenin) to escape internalization,^34^ with *β*-catenin and *α*-catenin being anchored to the actin cytoskeleton.^35^ Disruption of VEC-dependent AJs, for example, in response to inflammatory mediators such as lipopolysaccharide, induces the loss of endothelial integrity, accompanied by increased endothelial permeability.^36,37^ Null mutation of VEC leads to severe defects in junctional morphology and vascular morphogenesis, whereas partially knocking down VEC can increase vascular permeability.^38^ In addition, the vascular integrity of mice can be disrupted merely by blocking VEC with antibodies, which concomitantly increases permeability and hemorrhage.^39^ Taken together, inhibition of ROCK protected against vascular integrity-based cerebral hemorrhage, probably via the VEC signaling pathway. To validate this hypothesis, we used atorvastatin, a small-molecule inhibitor of HMG-CoA reductase. High-concentration atorvastatin can induce cerebral hemorrhage in a zebrafish model and disrupts the junction of ECs *in vitro*.^12,19,20^ Interestingly, we identified a new ROCK1 inhibitor, FPND, which had stronger protective effects than those of other ROCK inhibitors (for example, H1152) on atorvastatin-induced cerebral hemorrhage in zebrafish. To investigate the function of ROCK in cerebral hemorrhage, we examined its effects on the junction of ECs. As expected, the *in vitro* results showed that high-concentration atorvastatin significantly shrank ECs and decreased cell–cell junctions, effects that were reversed by FPND treatment. To further understand the effects of ROCK on the junction of ECs, we focused on the cytoskeleton arrangement and VEC signaling pathway after FPND treatment. FPND prevented atorvastatin-induced scrambled knots, assembly of focal adhesion complexes, formation of membrane ruffle close to cell–cell contacts, and drastic losses of VE-catenin, *β*-catenin and p120-catenin from cell borders, which marked the rupture of AJs. Phosphorylation of VEC leads to the uncoupling of p120- and *β*-catenin, internalization and ubiquitination of VEC, and disruption of cell–cell junctions.^40,41^ Partly reducing VEC expression may generate important defects in vascular integrity, such as cranial hemorrhages, and increase the vascular permeability in zebrafish embryos. In this study, statin-stimulated phosphorylation of MYPT1, LIMK1 and VEC was reversed by FPND treatment. H1152 and ROCK1 siRNA also decreased the phosphorylation of VEC, further suggesting that FPND prevented atorvastatin-induced cerebral hemorrhage through cytoskeletal rearrangement and enhancement of cell–cell junctions in ECs via the ROCK1 and VEC signaling pathways. Furthermore, FPND also prevented atorvastatin-induced hemorrhagic stroke and locomotor activity defects, and the mice could tolerate high-concentration FPND that was orally or intravenously administered. Collectively, FPND is a promising drug candidate for treating vascular integrity-based cerebral hemorrhage.

Although the relationship between the ROCK1 and VEC signaling pathways has been explored, further studies are required to determine whether the difference between the effects of ROCK1 inhibitor and the ROCK inhibitor H1152 on cerebral hemorrhage is linked to ROCK2. Furthermore, the interaction between ROCK1 and VEC still needs in-depth study.

In summary, FPND with a novel scaffold was able to inhibit ROCK1 specifically at a low micromolar concentration. It significantly protected against the cerebral hemorrhage induced by the loss of vascular endothelial integrity by regulating the ROCK and VEC signaling pathways. Moreover, the protective effects of FPND on vascular endothelial integrity were at least partially mediated through rearrangement of the actin cytoskeleton and enhancement of VEC-mediated cell–cell junctions. Given the extremely low toxicity of FPND against mice, FPND was safe. Our results support the use of FPND to prevent hemorrhagic stroke, particularly that associated with rupture of endothelial cell–cell junctions and deficiencies in vascular endothelial integrity.

### Significance

Using docking-based virtual screening and *in vitro* enzyme-based assays approach, we revealed a new compound (FPND) with a novel scaffold could inhibit ROCK1 specifically at low micromolar concentration. In *in vivo* studies, FPND significantly prevented statin-induced cerebral hemorrhage in a zebrafish model. In addition, *in vitro* studies showed that FPND prevented statin-induced cerebral hemorrhage by enhancing endothelial cell–cell junctions through inhibiting the ROCK-mediated VE-cadherin signaling pathway. As indicated by the extremely low toxicity of FPND against mice, it is safe and can potentially prevent vascular integrity loss-related diseases, such as hemorrhagic stroke.

## Materials and methods

### Ethics statement 

All animal experiments were conducted according to the ethical guidelines of the Institute of Chinese Medical Sciences (ICMS), University of Macau, and the protocol was approved by the Institute of Chinese Medical Sciences – Animal Ethics Committee (ICMS-AEC) of the University of Macau (permit number: 20120601).

### Cell culture and material

HUVECs were obtained from Thermo Fisher Scientific Inc. (Waltham, MA, USA) maintained in vascular cell basal medium (ATCC, Manassas, VA, USA) and used before to passage 7. Fetal bovine serum, phosphate-buffered saline (PBS), penicillin–streptomycin (PS) and 0.25% (w/v) trypsin/1 mM EDTA were all purchased from Invitrogen (Carlsbad, CA, USA). EC growth supplement, heparin, gelatin were supplied by Sigma (St Louis, MO, USA). Dimethyl sulfoxide (DMSO) and anti-VE-cadherin antibody, phospho [Tyr731] were also acquired from Sigma. Antibodies of ve-cadhrin, p-LIMK1, LIMK1, P-MYPT1, MYPT1, P-cofilin, cofilin and beta-actin were all purchased from Cell Signaling Technology (Danvers, MA, USA).

### Enzymatic inhibitory activity assays of ROCK

The enzymatic activity assays of lead compounds against ROCK were all conducted in 384-well plates by using the Z’-LYTE kinase assay kit (Thermo Fisher Scientific Inc.). In brief, 1.5 *μ*M peptide substrate, 5 ng ROCK1 or ROCK2 enzyme and testing compounds were added to each well and the reaction was then initiated by adding 12.5 *μ*M ATP. The plate was shaken on a plate shaker for 30 s and then incubated for 1 h at room temperature. Development solution was then added immediately and the assay plate was shaken for 30 s before incubation for another hour at room temperature. Stop reagent was then added to terminate the reaction. Finally, the coumarin (Ex. 400 nm, Em. 445 nm) and fluorescein (Ex. 400 nm, Em. 520 nm) emission signals were measured by the fluorescence plate reader. The inhibitory activity against ROCK1 or ROCK2 was quantified by calculating the IC_50_ values.

### Molecular docking

The structure of FPND was sketched and minimized by using the OPLS-2005 force field^42^ in Schrödinger (version 9.0) (Schrödinger, LLC, New York, NY, USA). The crystal structures of ROCK1 (PDB entry: 2ETR) and ROCK2 (PDB entry: 2H9V), retrieved from the RCSB Brookhaven Protein Data Bank, were used as the templates for molecular docking. The Protein Preparation wizard in Schrödinger 9.0 was used to remove all crystallographic water molecules, add hydrogen atoms, assign partial charges and minimize each structure until the root-mean-square deviation reached a maximum value of 0.3 Å. FPND was then docked into the active site of ROCK1 or ROCK2 by using the *Glide* module in Schrödinger with the extra precision (XP) scoring mode. The receptor grid box for glide docking was generated and centered on the ligand in the active site with a size of 10 Å×10 Å×10 Å×10 Å×10 Å.

### MDs simulations

The binding poses with the best docking scores were then submitted to the following MD simulations. FPND was optimized by the semi-empirical AM1 method in Gaussian09 followed by the single-point HF/6-31G* calculation of electrostatic potentials,^43^ and the partial charges and force field parameters for FPND were obtained using the *antechamber* program in AMBER11.^44^ Counter ions of Na^+^ were added to neutralize the charge of each system. Then, each system was immersed into a rectangular box of TIP3P water molecules, at a distance of 10 Å from any solute atom. The particle mesh Ewald (PME) method^45^ was used to handle long-range electrostatics in a periodic boundary condition. The general AMBER force field (*gaff*) and the ff99SB force field^46^ were used for FPND and the proteins, respectively.

Each system was optimized by two-stage minimization with the *sander* program in AMBER11^44^ before the MD simulations. In the first stage, the backbone carbons of each protein were constrained (50 kcal/mol/Å^2^) and each system was optimized by 1000 cycles of minimization (500 cycles of steepest descent and 500 cycles of conjugate gradient minimization). In the second stage, the whole system, with no constraint, was optimized by 5000 cycles of minimization (1000 cycles of steepest descent and 4000 cycles of conjugate gradient minimization). After that, each system was gradually heated from 0 to 300 K over a period of 50 ps followed by 5 ns NPT MD simulations with a target temperature of 300 K and a target pressure of 1 atm. The SHAKE algorithm was used to constrain all bonds involving hydrogen atoms.^47^ The time step was set as 2.0 fs and the coordinate trajectories were saved every 10 ps during the MD runs.

### MM/GBSA binding free energy calculations and decompositions

The binding free energy for each system was calculated by the MM/GBSA method, implemented in AMBER according to Equation 1:^48,49^

(1)ΔGbind=Gcomplex−Gprotein−Gligand=ΔH+ΔGsolvation−TΔS=ΔEMM+ΔGGB+ΔGSA−TΔS

where Δ*E*_MM_ represents the gas–phase interaction energy between protein and ligand, contributed from the electrostatic and van der Waals interactions; Δ*G*_*GB*_ is the polar component of the desolvation free energy; Δ*G*_*SA*_ is the non-polar component of the desolvation free energy; −*T*Δ*S* represents the change in conformational entropy upon ligand binding, which was ignored here due to the expensive computational cost and low prediction accuracy.^48,50^ Δ*G*_GB_ was estimated by using the generalized born (GB) model with the parameters developed by Onufriev*et al.* (*igb*=2).^51^ The exterior dielectric constant was set to 80, and the solute dielectric constant (*ε*_in_) was set to 2. Δ*G*_SA_ was calculated based on the solvent-accessible surface area (SASA), determined by the LCPO method.^52^ A total of 100 snapshots, evenly extracted from 3 to 5 ns, were used to calculate the energy terms.

The total protein–inhibitor interaction was decomposed into residue–inhibitor pairs by using the MM/GBSA decomposition protocol in AMBER^53,54,55^ The residue–inhibitor interactions consist of wan der Waals contributions (Δ*E*_vdw_), electrostatic contributions (Δ*E*_ele_), polar contributions of desolvation (Δ*G*_GB_) and non-polar contributions of desolvation (Δ*G*_SA_). Δ*G*_GB_ was estimated by the GB model with the parameters developed by Onufriev*et al.* (*igb*=2),^51^ and Δ*G*_SA_ was calculated based on SASA with the ICOSA technique.^55^

### Cell–cell junction detection using the xCELLigence system

The xCELLigence RTCA System (RTCA DP Station, Roche, San Diego, CA, USA) is a non-invasive, label-free platform that utilizes impedance changes across the cell monolayer to indirectly measure cell–cell interaction, transient contractions and cell layer permeability. ECs and the RTCA system (Roche) enable *in vitro* screening of compound effects on human endothelium permeability.^21,22,23^ For xCELLigence electrical conductivity assays, HUVECs were cultured and maintained on a 16-well E-Plate for 2 days to form a stable monolayer, pretreated with FPND, H1152 and/or PP2 for 2 h, and then treated with 2 *μ*M atorvastatin for 24 h. Data analysis of cell–cell junctions in HUVECs was accomplished with the RTCA software (Roche). Values represent the mean of triplicate points for all experiments.

### Immunofluorescence microscopy

Cells were seeded on 96-well plates, and mature junctions were allowed to establish over 2 days. When the experiment was done, cells were washed once for 1 min with PBS and fixed for 20 min with 4% formaldehyde in PBS. After being rinsed once with PBS, cells were permeabilized with PBS containing 0.3% Triton X-100 for 20 min on ice. After being washed three times with PBS for 1 min, cells were blocked with 2% BSA in PBS for 1 h at room temperature. Primary antibodies were applied in 2% BSA in PBS and incubated overnight at 4 °C. Cells were then washed three times for 1 min in PBS. Secondary antibodies conjugated to fluorescent probes (Alexa Fluor 488 rabbit anti-goat IgG (H+L), Molecular Probes, Thermo Fisher Scientific Inc.) were applied for 1 h at room temperature. Cells were washed 4× with PBS for 5 min per wash and images were taken with the IN Cell Analyzer 2000 system (General Electric, Marlborough, MA, USA).

### Western blotting analysis

Cells were pretreated with FPND for 2 h before the addition of 2 *μ*M atorvastatin at 15, 30 and 45 min. Cells receiving DMSO (0.1%) served as the vehicle control, which was equivalent to no treatment. Cells were then washed with PBS and lysed for 30 min on ice with lysis buffer (0.5 M NaCl, 50 mM Tris, 1 mM EDTA, 0.05% SDS, 0.5% Triton X-100, 1 mM PMSF, pH 7.4). Cell lysates were centrifuged at 11 000×*g* for 20 min at 4 °C. Protein concentrations in the supernatants were measured using the bicinchoninic acid assay (Pierce, Rockford, IL, USA). Supernatants were electrophoresed on 12% SDS-PAGE, and transferred to polyvinylidene diuoride membranes, which were then blocked with 5% non-fat milk. Immunoblot analysis was undertaken by incubating with antibodies at 4 °C overnight. After washing, membranes were incubated for 1 h at room temperature with horseradish peroxidase-conjugated goat anti-rabbit IgG. Proteins were detected using an advanced enhanced ECL system (GE Healthcare, Little Chalfont, UK). Semiquantifications were performed with densitometry analysis by Quantity One software (Quantity One, Hercules, CA, USA).

### Maintenance of zebrafish and embryos

Tg(*fli1a*:EGFP)y1; Tg(*gata1a*:dsRed)sd2 homozygous double transgenic zebrafish, which expresses green fluorescent protein (GFP) under the control of fli1 promoter in EC, and red fluorescent protein (dsRed) under the control of gata1 promoter in erythrocytes, were kindly provided by ZFIN (Eugene, OR, USA) and wild-type zebrafish was purchased from a local pet shop. The embryos were cultured at 28.58 °C in embryo medium that was prepared according to ZFIN’s instructions.

### Morphological observations of zebrafish

*Tg(fli1a:EGFP)y1; Tg(gata1a:dsRed)sd2* homozygous double transgenic zebrafish embryos at 21 h post-fertilization (hpf) were pretreated with different concentrations of both FPND (1, 3, 10, 30 or 100 *μ*M) and H1152 (1.25, 2.5 or 5 *μ*M) for 3 h, whereas the embryos treated with 0.2% DMSO (solvent) for 3 h served as the vehicle control group. Then, the pretreated embryos were treated with 2 *μ*M atorvastatin for 24 h and observed for viability and gross morphological changes under a fluorescence microscope (Olympus MVX10; Tokyo, Japan) equipped with a digital camera (ColorView II, Soft Imaging System; Olympus). The images were analyzed with Adobe Photoshop 7.0 and ImageJ software (ImageJ, Bethesda, MD, USA). To evaluate hemorrhage stroke in zebrafish embryos, the indexes of hemorrhage were quantified by measuring the area of hemorrhage in cerebra.

### Preparation of FPND solution for intravenous injection (i.v. injection)

A certain amount of Solutol HS 15 was heated up to 60 °C in a water bath for dissolving. Then, 1 ml dissolved Solutol was added into the glass tube with 2.5 mg FPND. Heat and ultrasound methods were used until the drug completely dissolved. Then, 4 ml water was added into the tube and mixed thoroughly to obtain 1 mg/ml FPND solution.

### Acute toxicity test for FPND

Mature BalB/c mice with a minimum body weight of 20 g were used, and the concentration administered orally was 10 mg/kg of body weight, and i.v. 0.5 mg/kg of body weight. At the target dose, all mice should be alive for 2 weeks. During the 2-week period, the weight of each mouse was recorded every 2 days. All experiments were in compliance with national regulations on the administration of experimental animals, approved by The Hong Kong Polytechnic University (license key: SCXK 2008-0002; 44007200004512).

### Statistical analysis

Statistical analysis was performed using PRISM software (version 5.0, GraphPad Software, La Jolla, CA, USA). All experiments were performed at least in triplicate. Data are expressed as means±S.D. Statistical testing included one-way ANOVA and Student’s *t*-test, applied as appropriate and with *P*<0.05 considered statistically significant.

## Publisher’s note

Springer Nature remains neutral with regard to jurisdictional claims in published maps and institutional affiliations.

## Figures and Tables

**Table 1:** The predicted binding free energies and the individual energy components of FPND toward ROCK1 and ROCK2 (*ε*_in_=2.0)

*Ligand*	*Protein*	*Non-polar contributions*		*Polar contributions*		*Δ*G**_*pred*_
		*Δ*E**_*vdw*_	*Δ*G**_*SA*_	*Δ*E**_*ele*_	*Δ*G**_*GB*_	
FPND	ROCK1	−51.80±2.92	−4.53±0.12	−183.37±3.06	189.14±2.00	−50.57±1.97
FPND	ROCK2	−34.88±0.03	−3.33±0.03	−171.43±5.3	176.58±4.84	−34.88±0.03
