# Supplementary material for: Discovery of a ROCK inhibitor, FPND, which prevents cerebral hemorrhage through maintaining vascular integrity by interference with VE-cadherin
Source: Cell Death Discov. 2017 Aug 21;3:17051–. doi: 10.1038/cddiscovery.2017.51 (PMC5563523; doi:10.1038/cddiscovery.2017.51)
Supplement: Supplementary Information [file cddiscovery201751-s1.docx]

**Supporting Materials**


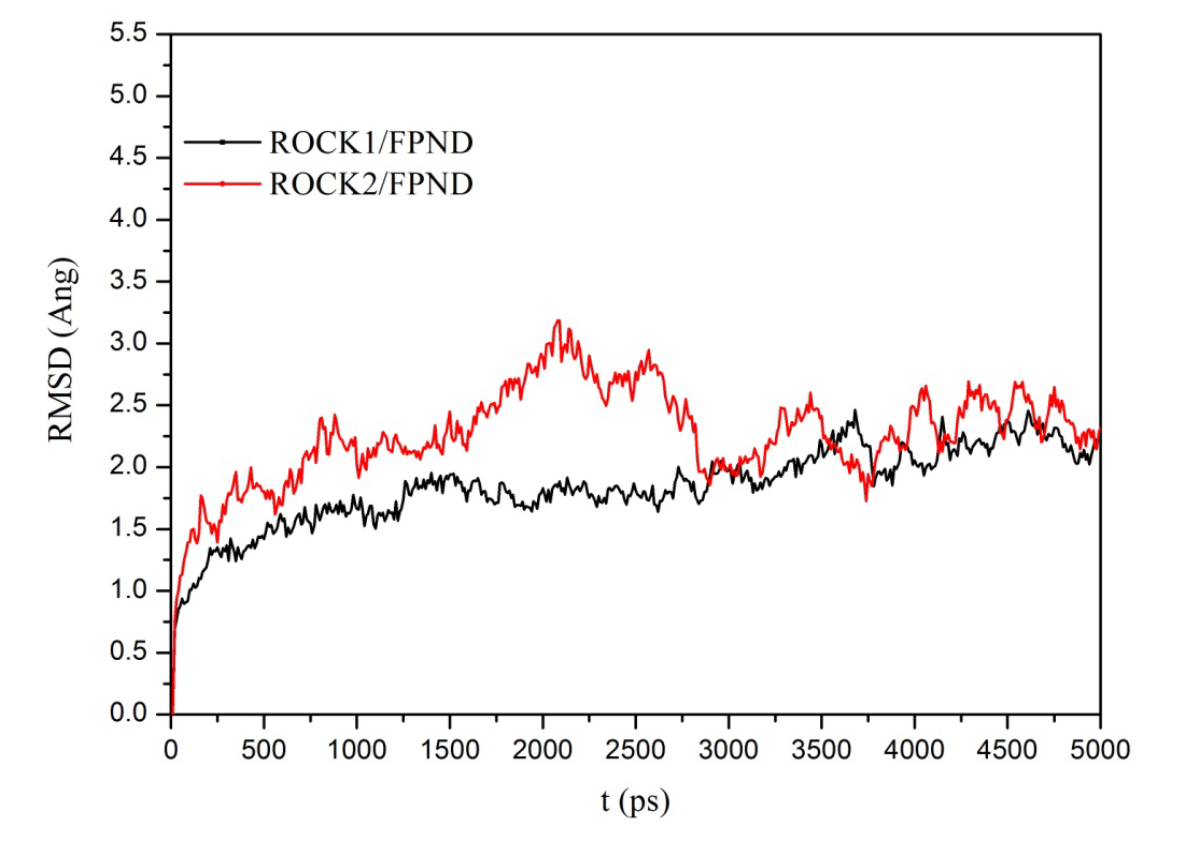


**Figure S1**. Root-mean-square displacement (RMSD) of the backbone Cα atoms of the ROCK1/FPND complex and ROCK2/FPND complex with respect to the first snapshot as a function of time.


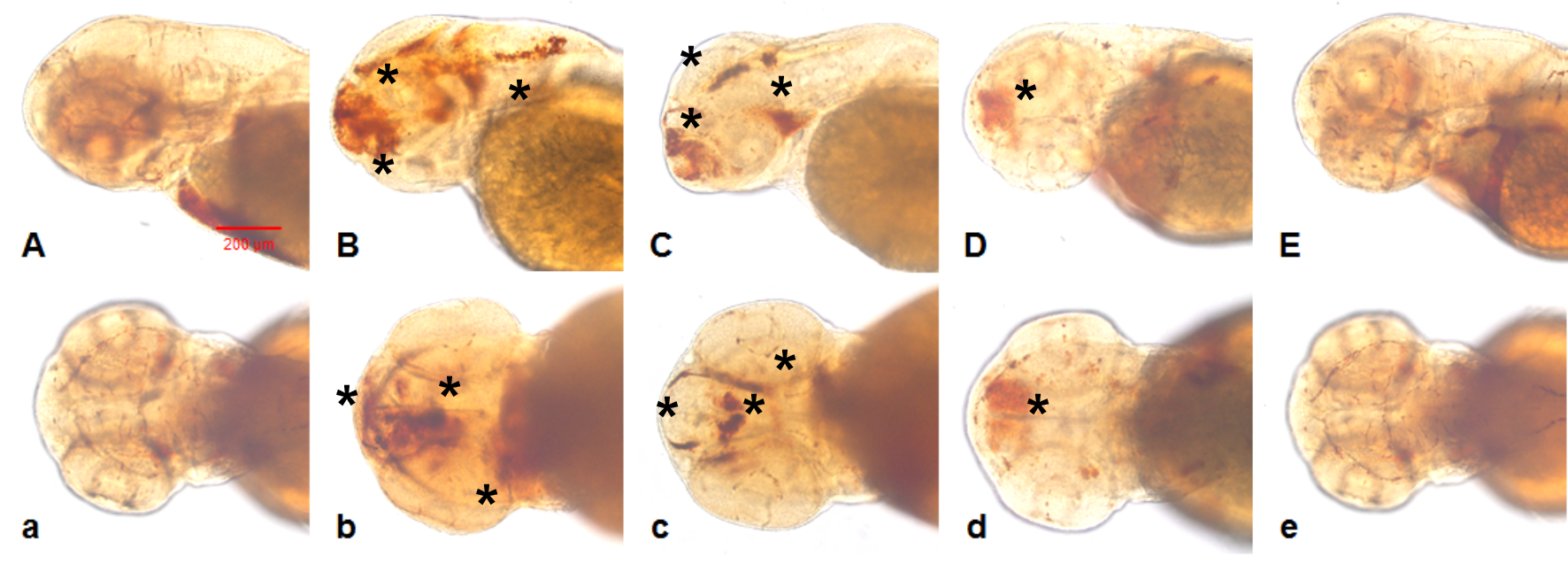


**Figure S2**. FPND prevented atorvastatin-induced zebrafish cerebral hemorrhage as shown by o-dianizidine staining. The 22 dpf embryos were pretreated with either 0.1% DMSO (solvent) (A, a, B, b), 10 (C, c), 30 (D, d) or 100 μM (E, e) FPND for 3 hours and replaced with 0.1% DMSO (A, a) or 2 μM atorvastatin (B-E, b-E) for 24 h. The embryos were treated with 0.1% DMSO (solvent) served as normal control group (A). The asterisks indicate the erythrocyte accumulation in cerebral hemorrhage region in zebrafish head. Red scale bar represents 200 μm.


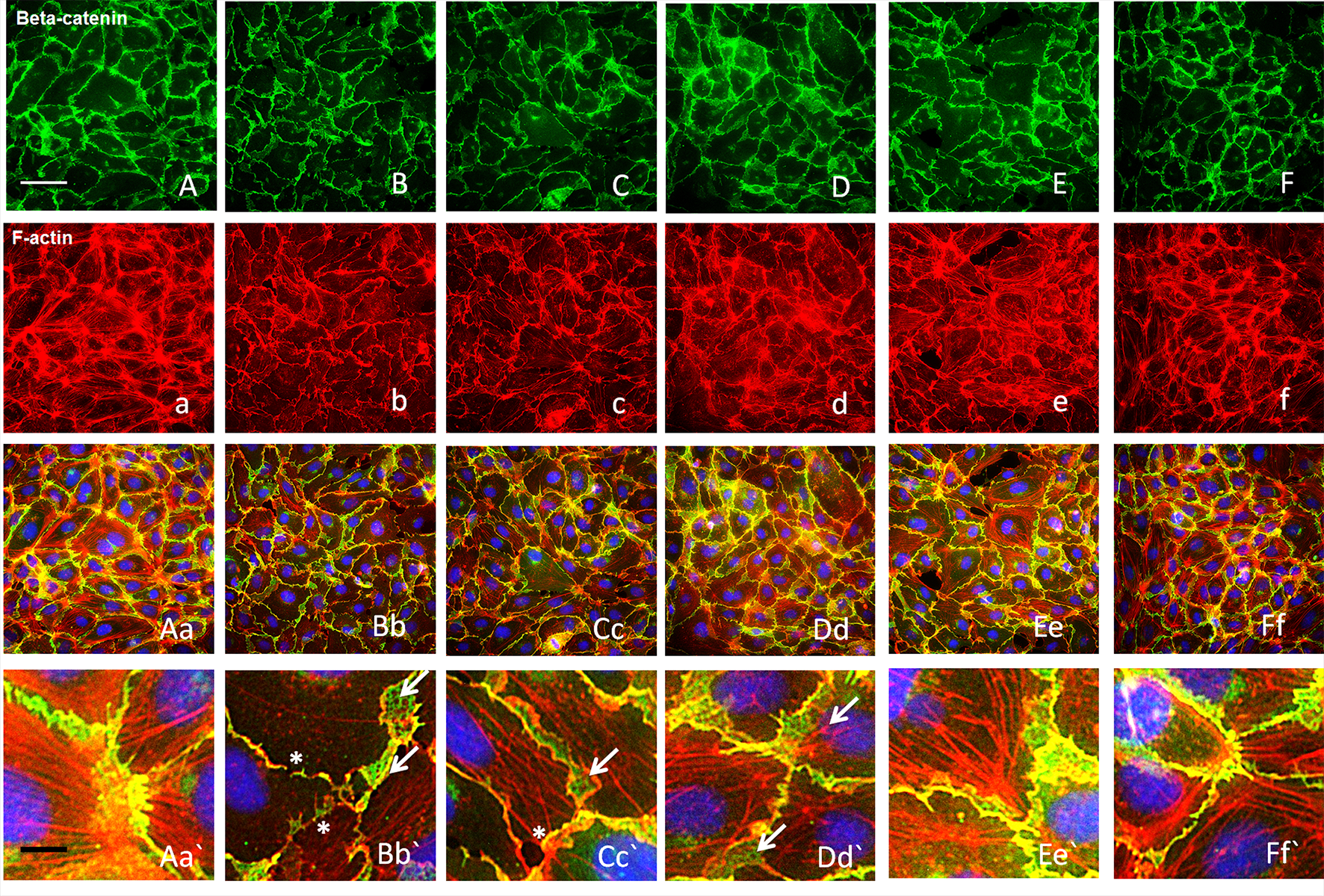


**Figure S3**. FPND prevents atorvastatin damaged cell-cell junction and decreased β-catenin distribution in cell-cell junctions regions. HUVECs monolayers were treated with 0.1% DMSO (A, a, Aa, A`; B, b, Bb, B`), 5 (C, c, Cc, Cc`), 10 (D, d, Dd, Dd`), 20 (E, e, Ee, Ee`; F, f, Ff, Ff`) μM FPND for 2 hours then washout and treated with 0.1% DMSO (A, a, Aa, A`; f, Ff, Ff`) or 2 μM atorvastatin (B, b, Bb, B`; C, c, Cc, Cc`; D, d, Dd, Dd`; E, e, Ee, Ee`) for 12 hours. 0.1% DMSO treated for 12 h as a vehicle control (A, a, Aa, Aa`). Treatment of FPND alone (F, f, Ff, Ff`) shows slightly decreased stress fiber formation but no affection of β-catenin distribution and cell-cell junctions. β-catenin signal was labeled with β-catenin specific antibody in green color (A-F). F-actin was labeled with tetramethyl rhodamine isothiocyanate (TRITC)-phalloidin in red color and nucleis were labeled with nuclear specific dye Hochest 33342 in blue color (a-f). White asterisks indicated scrambled knots, membrane ruffle and focal adhesion complexes assemble formation. White arrows showed a drastic loss of β-catenin from cell borders and formed a net-like structure. White and black scale bar represents 50 μm and 10 μm, respectively.


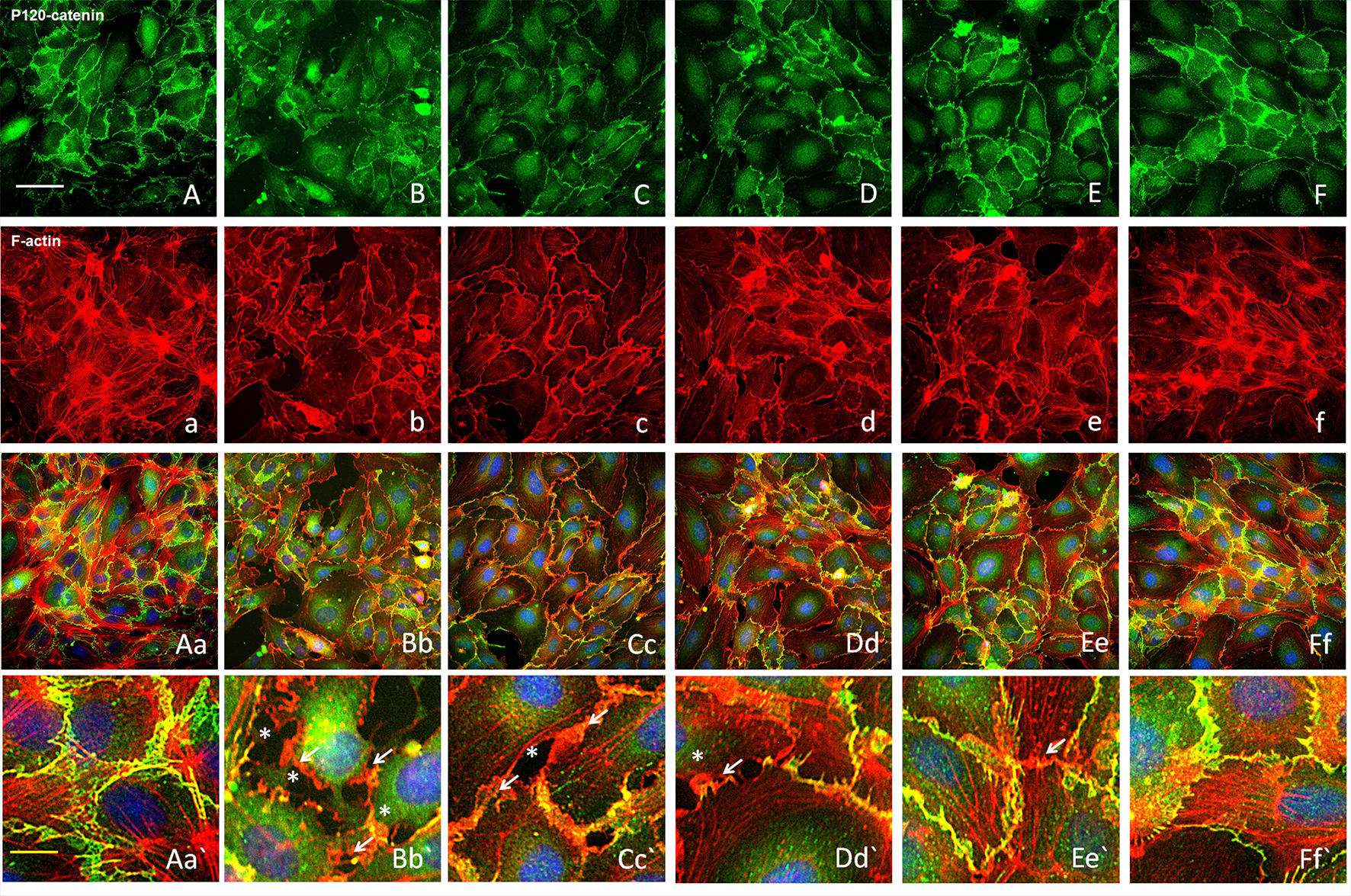


**Figure S4**. FPND prevents atorvastatin damaged cell-cell junction and decreased p120-catenin distribution in cell-cell junctions regions. HUVECs monolayers were treated with 0.1% DMSO (A, a, Aa, A`; B, b, Bb, B`), 5 (C, c, Cc, Cc`), 10 (D, d, Dd, Dd`), 20 (E, e, Ee, Ee`; F, f, Ff, Ff`) μM FPND for 2 hours then washout and treated with 0.1% DMSO (A, a, Aa, A`; f, Ff, Ff`) or 2 μM atorvastatin (B, b, Bb, B`; C, c, Cc, Cc`; D, d, Dd, Dd`; E, e, Ee, Ee`) for 12 hours. 0.1% DMSO treated for 12 hours as a vehicle control (A, a, Aa, Aa`). Treatment of FPND alone (F, f, Ff, Ff`) shows slightly decreased stress fiber formation but no affection of p120-catenin distribution and cell-cell junctions. P120-catenin signal was labeled with p120-catenin specific antibody in green color (A-F). F-actin was labeled with tetramethyl rhodamine isothiocyanate (TRITC)-phalloidin in red color and nucleis were labeled with nuclear specific dye Hochest 33342 in blue color (a-f). White asterisks indicated scrambled knots, membrane ruffle and focal adhesion complexes assemble formation. White arrows showed a drastic loss of p120-catenin from cell borders and formed a net-like structure. White and yellow scale bar represents 50 μm and 10 μm, respectively.


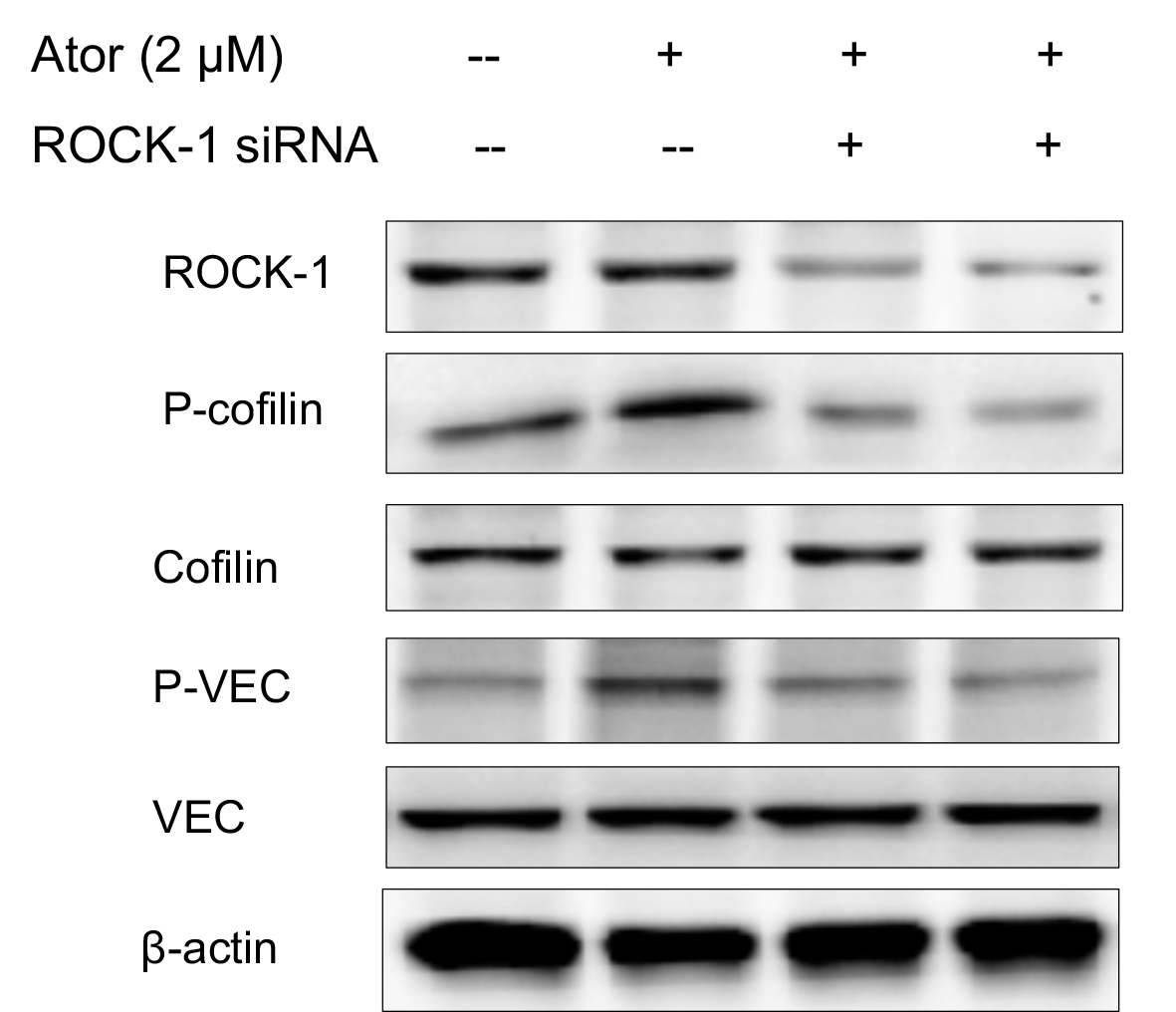


**Figure S5**. Knockdown of ROCK-1 reduced phosphorylation of VEC. HUVEC cells were transferred with control siRNA or ROCK-1 siRNA for 48h and then stimulated with 2 µM atorvastatin for 30 min. (A) The expression ratio of phosphorylated VEC/total VEC, phosphorylated confilin/total confilin, ROCK-1 and β-actin, were detected by Western blotting with specific indicated antibodies as indicated.


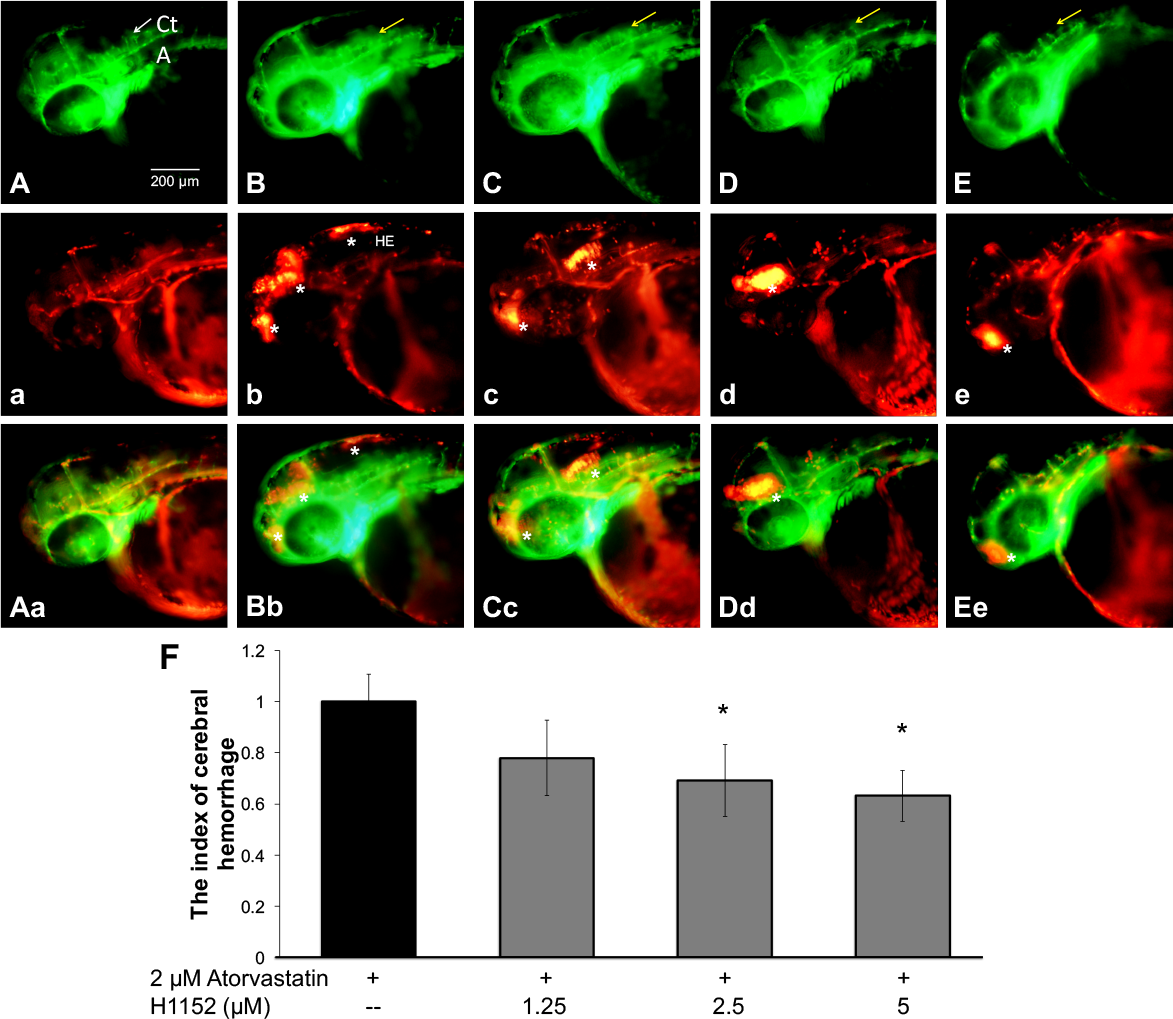


**Figure S6**. The effect of ROCK inhibitors in atorvastatin induced zebrafish cerebral hemorrhage. (A) 1 dpf embryos were treated with 0.2% DMSO (solvent) served as normal control group; (B, C, D, E) The 1 dpf embryos were pretreated with either 0.2% DMSO (solvent), 1.25, 2.5 and 5 μM H-1152 for 3 hours and replaced with 2 μM atorvastatin for 24 h. The orange color arrows indicated the erythrocyte accumulation in cerebral hemorrhage region in zebrafish head. (F) The representative cell index indicated H-1152 significantly prevented atorvastatin-induced cerebral hemorrhage in zebrafish. **P<0.05* versus control groups were considered significantly different.
